# Supplementary figures and images for: Evolution analysis of FRIZZY PANICLE (FZP) orthologs explored the mutations in DNA coding sequences in the grass family (Poaceae)
Source: PeerJ. 2022 Mar 11;10:e12880. doi: 10.7717/peerj.12880 (PMC8919851; doi:10.7717/peerj.12880)

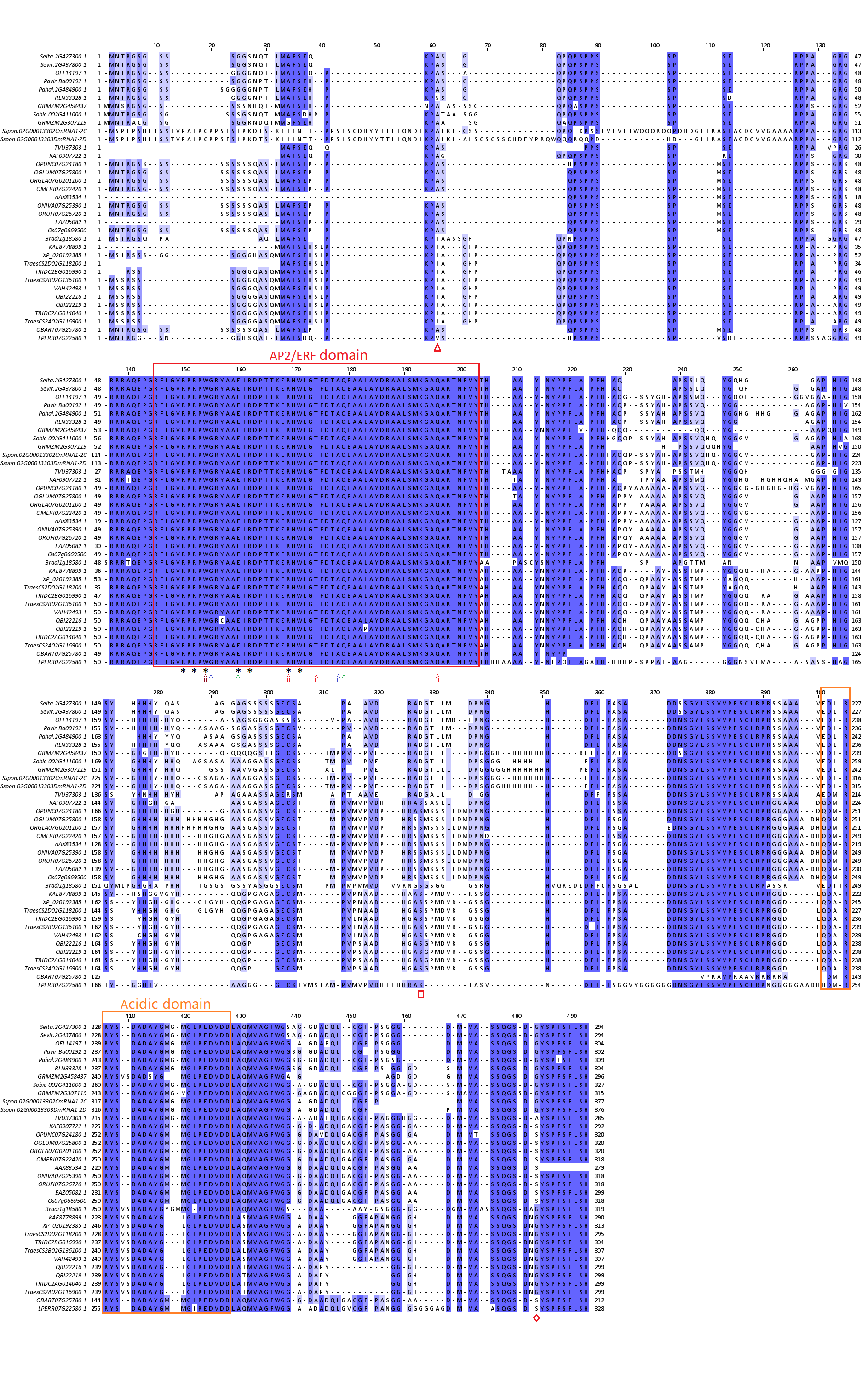

Supplement: Figure S1 — The red and orange frames indicate AP2/ERF domain and acidic domain of FZP orthologs gene. Amino acids residues with asterisks indicate that they confer specific GCC box binding. The amino acid residues with brown, blue, green and red arrows indicate mutation sites that can cause supernumerary spikelets (SSs) phenotype in maize, Brachypodiumdistachyon, bread wheat and rice, respectively. An amino acid substitution is shown at positions 157 (Y157C: Y at position 157 is changed to C) and 183 (L183P: L at position 39 is changed to P) of the QBI22216.1 and QBI22219.1 genes, respectively. The amino acid residues with red triangle, diamond and box indicate significant positive selection sites in a branch of Triticeae, Oryza, and C4 plants, respectively. [file peerj-10-12880-s001.png]

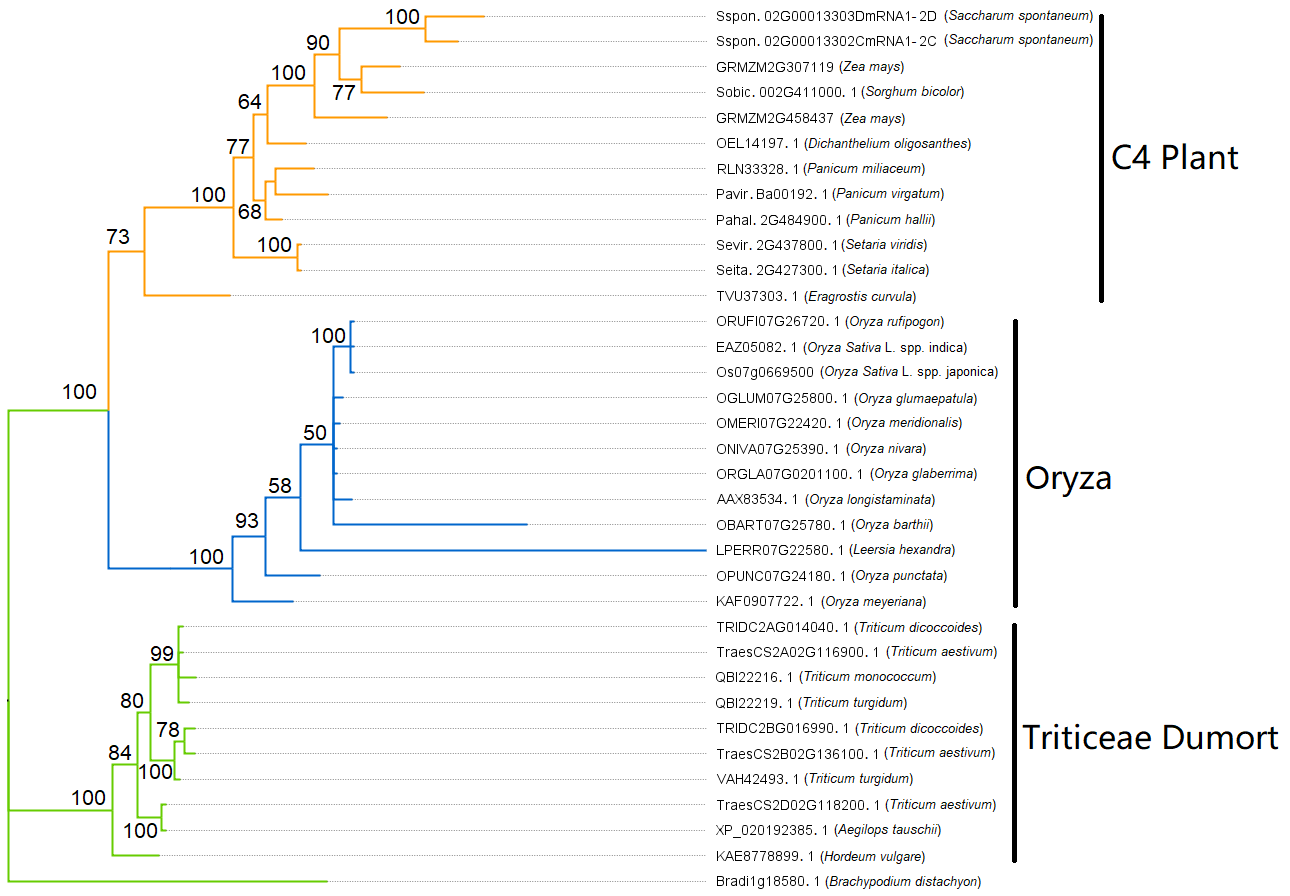

Supplement: Figure S2 — The numbers above the branches indicate the posterior probability. The branch labels are tagged with gene ID numbers and Latin names of species (inside brackets). [file peerj-10-12880-s002.png]
