# Supplementary material for: Evolution analysis of FRIZZY PANICLE (FZP) orthologs explored the mutations in DNA coding sequences in the grass family (Poaceae)
Source: PeerJ. 2022 Mar 11;10:e12880. doi: 10.7717/peerj.12880 (PMC8919851; doi:10.7717/peerj.12880)
Supplement: Supplemental Information 3 — a The confidence of each model is quantitatively measured by C-score that is calculated based on the significance of threading template alignments and the convergence parameters of the structure assembly simulations. C-score is typically in the range of [ −5, 2], where a C-score of a higher value signifies a model with a higher confidence and vice-versa (https://zhanggroup.org/I-TASSER/). b, c TM-score and RMSD are estimated based on C-score and protein length following the correlation observed between these qualities. Since the top 5 models are ranked by the cluster size, it is possible that the lower-rank models have a higher C-score in rare cases. The first model has a better quality in most cases (https://zhanggroup.org/I-TASSER/). [file peerj-10-12880-s003.docx]

Table S1: The parameters of the top 5 final models of the 5 genes predicted by I-TASSER

| **Gene ID** | **Model 1** | | | **Model 2** | **Model 3** | **Model 4** | **Model 5** |
| --- | --- | --- | --- | --- | --- | --- | --- |
|  | **C-score^a^** | **Estimated TM-score^b^** | **Estimated RMSD^c^** | **C-score** | | | |
| Os07g0669500 | -2.54 | 0.42±0.14 | 12.2±4.4Å | -3.6 | -4.63 | -3.36 | -3.69 |
| TraesCS2A02G116900.1 | -2.77 | 0.4±0.13 | 12.8±4.2Å | -4.18 | -3.74 | -3.8 | -3.82 |
| GRMZM2G307119 | -2.44 | 0.43±0.14 | 12.1±4.4Å | -4.66 | -3.59 | -3.43 | -3.78 |
| QBI22216.1 | -3.53 | 0.33±0.11 | 14.8±3.6Å | -4.32 | -4.02 | -3.47 | -4.23 |
| QBI22219.1 | -2.74 | 0.4±0.13 | 12.7±4.3Å | -4.33 | -4.37 | -4.06 | -3.55 |

^a^ The confidence of each model is quantitatively measured by C-score that is calculated based on the significance of threading template alignments and the convergence parameters of the structure assembly simulations. C-score is typically in the range of [-5, 2], where a C-score of a higher value signifies a model with a higher confidence and vice-versa (https://zhanggroup.org/I-TASSER/).

^b, c^ TM-score and RMSD are estimated based on C-score and protein length following the correlation observed between these qualities. Since the top 5 models are ranked by the cluster size, it is possible that the lower-rank models have a higher C-score in rare cases. The first model has a better quality in most cases (https://zhanggroup.org/I-TASSER/).
